# Intimate partner sexual violence and risk for femicide, suicidality and substance use among women in antenatal care and general out-patients in Thailand

**DOI:** 10.1186/s12905-018-0526-z

**Published:** 2018-02-06

**Authors:** Supa Pengpid, Karl Peltzer, Orapin Laosee, Kawinarat Suthisukon

**Affiliations:** 1 ASEAN Institute for Health Development, Mahidol University, Salaya, Phutthamonthon, Nakhonpathom, 73170 Thailand; 2 Department of Research & Innovation, University of Limpopo, Sovenga, 0727 South Africa; 3 HIV/AIDS/STI/and TB (HAST), Human Sciences Research Council, Pretoria, 0001 South Africa

**Keywords:** Sexual assault, Psychological abuse, Physical violence, Danger, Intimate partner, Thailand

## Abstract

**Background:**

Little is known about the occurrence and health consequences of intimate partner sexual assault. The aim of this study was to assess the prevalence and correlates of sexual assault in the context of intimate partner violence (IPV) in Thailand.

**Methods:**

In a cross-sectional survey adult female participants were systematically screened (self-administered or interview administered) for IPV in antenatal care and general outpatient clinics in nine randomly selected hospitals in two provinces in the central region. Measures included the Abuse Assessment Screen, Severity of Violence Against Women Scale, Danger assessment and suicidal behaviour.

**Results:**

From 14,288 women screened, 1.5% were positive for IPV and 207 participated in the study. The mean age of the study participants was 26.8 years (SD = 9.3). Fifty-seven women, 27.5% of the sample, reported sexual assault, one or more times, during the relationship in the past 12 months. Most reported some form of psychological abuse (82.1%), physical violence (67.1%) and danger (72.0%). In all, 21.3% reported psychological, physical and sexual violence. Bivariate analyses found that older age, being recruited in the general out-patient department, greater number of children, high psychological abuse, high physical violence, danger and suicidal behaviour in the past 12 months were associated with sexual assault. In multivariable backward conditional logistic regression physical violence (OR = 5.32, CI = 2.52–11.24) and suicidal behaviour (OR = 3.28, CI = 1.37–7.83) were found to be associated with sexual assault.

**Conclusions:**

The study found a moderate rate of sexual assault in intimate violent partner relationships and those sexual assaults are more likely to co-occur with physical intimate partner violence and suicidal behaviour. This knowledge may be helpful in the detection and management of sexual assault in intimate violent partner relationships of women in health care settings in Thailand.

## Background

“Intimate partner violence refers to any behaviour within an intimate relationship that causes physical, psychological or sexual harm to those in that relationship” [1]. The prevalence and health effects of physical intimate partner violence (IPV) have been well documented, while less is known about the occurrence and health consequences of sexual IPV [2]. Globally, in studies (as reviewed in [3]) that measured sexual assault separately from physical violence, a high proportion of women (40**–**68%) had experienced both physical and sexual violence. Further, studies (as reviewed in [3]) consistently showed that compared to women who experienced physical intimate partner violence (only), women with sexual partner violence or sexual and physical partner violence had higher levels of physical and emotional intimate partner violence, had more risk factors for femicide, were more likely to report threatening or attempting suicide, used alcohol or substances and were not living with the abuser.

Among six European countries women in an antenatal care setting reported 2.7% emotional abuse in the past 12 months, 2.2% physical abuse, 0.4% sexual abuse and 3.8% any abuse in the past 12 months [4]. Past year intimate partner violence during pregnancy was 2.0% in Cambodia, 0.4% in the Philippines, 1.2% in Japan and 3.8% in rural and 4.2% in urban Thailand [5]. Other local surveys in Thailand found among pregnant women that 4.8% had experienced physical intimate partner violence during pregnancy and 4.8% sexual violence in the past 12 months [6], and in another study Saito et al. [7] found among prenatal women that 26.6% were exposed to overall threats and acts of physical violence during their current pregnancy, and Saito et al. [8] found among post-partum women that overall 9.5% were exposed to threats and physical violence and 11.3% sexual violence. There is a lack of studies on the prevalence and correlates of sexual assault in the context of intimate partner violence in Thailand.

The aim of this study was to assess the prevalence and correlates of sexual assault in the context of intimate partner violence in a sample of women attending antenatal care or general out-patient hospital services in Thailand.

## Methods

### Sample and procedure

Adult female participants were systematically screened for intimate partner violence in nine randomly selected hospitals (antenatal care and out-patient clinics) in Central Thailand. A recruiter to determine eligibility approached all women who presented to a study site for a health care visit using the Abuse Assessment Screen [9]. The screen included two questions, “1) During the last 12 months, have you been pushed, shoved, slapped, hit, kicked or otherwise physically hurt by someone? 2) During the last 12 months, have you been forced into sexual activities by someone?” [9] Response options were “yes” or “no”, and if yes, by whom. Written informed consent was obtained from all study participants who met the following inclusion criteria: (1) be female, (2) be 18 years of age and older, (3) have experienced IPV in the past 12 months, and (4) willingness to provide informed consent. Following an informed consent procedure, the interviewer verbally administered a questionnaire in Thai language in a private room without the partner or other individuals being present. All instruments were translated from English into Thai using standard backward and forward methods. The trained research assistants adhered strictly to the research principles of conducting research on violence against women [10], such as the safety of respondents and the researcher, and protecting confidentiality to ensure women’s safety and data quality. Participants were assured that their responses would be confidential and anonymous and that refusal would not jeopardise their management. For further management, women were referred to the hospital One-Stop Crisis Centre (OSCC). The study was conducted from November 2014 to October 2015.

### Measures

*Intimate partner violence* was assessed with 46 items of the *Severity of Violence Against Women Scale* (SVAWS); 19 items on threats of physical violence (scores 0 to 57), 21 items on physical assault (0 to 81 scores) and 6 items on sexual violence (scores 0 to 18) in the past 12 months [11, 12]. The SVAWS has been pilot tested for face validity in Thailand before [13]. (Cronbach alpha was 0.96).

The *Danger Assessment Scale* (15 items) was used to measure risk factors associated with homicide in situations including abuse in the past 12 months [14]; the sexual assault item was deleted from this scale, as it is covered by the SVAWS, so that the possible total range of scores was from 0 to 14. (Cronbach alpha was 0.88.

*Suicidal behaviour* was measured in terms of women reporting threatening or trying to commit suicide within the last 12 months [2].

The *Alcohol Use Disorder Identification Test– Consumption (AUDIT-C)* [15] was used to classify hazardous alcohol use or having an active alcohol use disorder [15]. (Cronbach alpha was 0.90).

*Sociodemographic* items assessed included, age, marital status, living with partner, education, number of children, employment status and subjective economic household situation.

### Data analysis

The data were analysed using IBM SPSS (version 22.0) (Chicago, IL, USA). Frequencies, means, standard deviations, were calculated to describe the sample. Data were checked for normality distribution and outliers, and for non-normally distributed data, non-parametric tests were used. For the logistic regression analysis, two groups were formed: 1) women who were sexually assaulted and 2) women who were never sexually assaulted by the intimate partner in the past 12 months. Adjusted odds ratios and 95% confidence intervals were calculated from multiple backward conditional logistic regression models to examine associations between sexual assault and demographic and partnership characteristics, other types of intimate partner violence, alcohol use and suicidal behaviour [3].

## Results

### Sample characteristics

In all, 14,288 women (3779 in antenatal care and 10,409 in general out-patient clinics) in nine hospitals were screened for IPV over a period of 9 months. From the 14,288 women screened, 212 screened positive for IPV (1.5%), of which five refused to be part of the study, so that the final sample was 207. The mean age of the study participants was 26.8 years (SD = 9.3). All were Thai, they had on average 1.1 children (SD = 1. 0), and most (73.8%) had a secondary or higher education. Most (63.2%) of the women had a good economic household situation (“We have most of the important things but few luxury goods.” Or “Some money for extra things such as going away for holidays and luxury goods.”).

Regarding participants’ relationships with their abusive partners, 87.0% were married or cohabiting, and 84.4% were living with their abusive partner at the time of the assessment and in 8.7% of the cases they had left their partner in the past 12 months. Fifty-seven women, 27.5% of the sample, reported sexual assault, one or more times, during the relationship in the past 12 months. Most reported some form of psychological abuse (82.1%), physical violence (67.1%) and danger (72.0%). In all, 21.3% reported psychological, physical and sexual violence. Thirty six (17.6%) of the women reported threatening or trying to commit suicide in the past 12 months and 23.3% have risky alcohol use in the past 12 months. More than a quarter (27.5%) reported sexual violence in the past 12 months (see Table 1).

**Table 1:** Sample characteristics

Variable	Total sample (*N* = 207)		Not sexually assaulted (*n* = 150,72.5%)	Sexually assaulted (*n* = 57, 27.5%)	*P*-Value
	M	SD	M (SD)	M (SD)	
Age (range 18–49)	26.8	9.3	25.9 (9.2)	29.1 (9.4)	0.019
Number of children (range 0–4)	1.1	1.0	0.9 (1.0)	1.4 (1.0)	0.005
Formal education					
Primary or less	54	26.2	35 (23.3)	19 (33.9)	0.253
Secondary	109	52.9	84 (56.0)	25 (44.6)	
Post secondary	43	20.9	31 (20.7)	12 (21.4)	
Recruitment					
General out-patient clinic	69	33.3	41 (59.4)	28 (40.6)	0.003
Antenatal care clinic	138	66.7	109 (79.0)	29 (21.0)	
Currently employed	89	51.1	64 (49.6)	25 (55.6)	0.492
Economic household situation					
Low	75	36.8	55 (37.2)	20 (35.7)	0.848
High	129	63.2	93 (62.8)	36 (64.3)	
Marital status					
Married/cohabitating	180	87.0	133 (88.7)	47 (82.5)	0.236
Single/divorced/separated	27	13.0	17 (11.3)	10 (17.5)	
Currently living with perpetrator	168	84.4	126 (85.7)	42 (80.8)	0.398
Left partner in the past 12 months	18	8.7	11 (7.3)	7 (12.3)	0.275
Other types of intimate partner violence					
Psychological abuse					
Low (0–2)	76	36.7	64 (84.2)	12 (15.8)	< 0.001
Medium (3–6)	64	30.9	54 (84.4)	10 (15.6)	
High (7 or more)	67	32.4	32 (47.8)	35 (52.2)	
Physical violence					
Low (0–1)	86	41.7	73 (84.9)	13 (15.1)	< 0.001
Medium (2–6)	53	25.7	40 (75.5)	13 (24.3)	
High (7 or more)	67	32.5	37 (55.2)	30 (44.8)	
Danger					
Low (0–1)	112	54.1	95 (63.3)	17 (29.8)	< 0.001
Medium (2–3)	36	17.4	26 (17.3)	10 (17.5)	
High (4 or more)	59	28.5	29 (19.3)	30 (52.6)	
Suicidal behaviour and alcohol use					
Suicidal behaviour (threat or attempt) in the past 12 months	36	17.6	16 (10.8)	20 (35.7)	< 0.001

### Types and frequency of sexual assault

More than half (57.9%) of the women reporting sexual assault had been forced to have sex against their will and 87.7% had been demanded to have sex whether they wanted to or not in the past 12 months (see Table 2).

**Table 2:** Types and frequency of sexual assault among sexually assaulted women (*N* = 57) in the past 12 months

Sexual assault	Once or more	Once	2–3 times	4 or more times
	N (%)	N (%)	N (%)	N (%)
1. Demanded sex whether you wanted to or not?	50 (87.7)	17 (29.8)	14 (24.6)	19 (14.0)
2. Made you have oral sex against your will?	18 (31.6)	4 (7.0)	8 (14.0)	6 (10.5)
3. Made you have sexual intercourse against your will?	33 (57.9)	9 (15.8)	12 (21.1)	12 (21.1)
4. Physically forced you to have sex?	24 (42.1)	7 (12.3)	6 (10.5)	11 (19.3)
5. Made you have anal sex against your will?	17 (29.7)	7 (12.3)	6 (10.5)	4 (7.0)
6. Used an object on you in a sexual way?	6 (10.5)	3 (5.3)	3 (5.3)	0 (0.0)

### Sexual assault and danger

Tables 3 shows the frequency of each risk factor for danger scores, along with the relative risk for sexually assaulted compared to non-sexually assaulted women. Sexually assaulted women reported statistically significant more risk factors of danger compared to not sexually assaulted women (*P* < 0.001). Regarding danger items, increase in severity or frequency of physical violence (Relative Risk Ratio, RRR = 1.47, 95% Confidence Interval, CI = 1.14–1.91), the use of a weapon (RRR = 1.92, CI = 1.18–3.13), threat to kill (RRR = 1.35, CI = 1.08–1.70), choking (RRR = 1.42, CI = 1.04–1.93), violently and constantly jealous (RRR = 1.26, CI = 1.10–1.68), control of daily activities (RRR = 1.59, CI = 1.18–2.13), spy on you (RRR = 1.48,CI = 1.17–1.87), and having been beaten while pregnant (RRR = 1.28, CI = 1.00–1.65) were found to be more frequent among sexually assaulted compared to non-sexually assaulted women (see Table 3).

**Table 3:** Frequencies of not sexually abused and sexually abused women who reported “Yes” to risk factor for danger within preceding 12 months days and Relative Risk (95% Confidence Interval)

By sexual abuse group	Not sexually assaulted (*N* = 150)	Sexually assaulted (*N* = 57)	Relative Risk Ratio (95% Confidence Interval)
	N (%)	N (%)	
1. Has the physical violence increased in severity or frequency in the past 12 months?	29 (19.3)	25 (43.9)	1.47 (1.14–1.91)*
2. Does he own a gun?	13 (8.7)	5 (8.8)	1.00 (0.74–1.35)
3. Has he ever used a weapon against you or threatened you with a lethal weapon 12 months?	10 (6.7)	15 (26.3)	1.92 (1.18–3.13)*
4. Does he threaten to kill you?	36 (24.0)	26 (45.6)	1.35 (1.08–1.70)*
5. Has he avoided being arrested for domestic violence in the past 12 months?	8 (5.3)	7 (12.3)	1.38 (0.86–2.24)
6. Does he ever try to choke you?	20 (13.3)	17 (29.8)	1.42 (1.04–1.93)*
7. Does he use illegal drugs in the past 12 months? By drugs, I mean “marihuana” or street drugs such as amphetamines (‘ya-baa’), ecstasy (‘ya E’), cocaine, “crack”	13 (8.7)	11 (19.3)	1.38 (0.95–2.02)
8. Is he an alcoholic or problem drinker?	44 (29.3)	18 (31.6)	1.03 (0.85–1.24)
9. Does he control most or all of your daily activities in the past 12 months?	24 (16.0)	24 (42.1)	1.59 (1.18–2.13)*
10. Is he violently and constantly jealous of you?	41 (27.3)	29 (50.9)	1.26 (1.10–1.68)*
11. Have you ever been beaten by him while you were pregnant in the past 12 months?	28 (18.7)	19 (33.3)	1.28 (1.00–1.65)*
12. Has he ever threatened or tried to commit suicide in the past 12 months?	9 (6.0)	4 (7.0)	1.05 (0.72–1.52)
13. Does he threaten to harm your children?	9 (6.0)	7 (12.3)	1.31 (0.85–2.04)
14. Does he follow or spy on you, leaves threatening notes or messages, destroys your property, or calls you when you don’t want him to?	36 (24.0)	30 (52.6)	1.48 (1.17–1.87)*
Danger scale, M (SD)	2.1 (3.1)	4.2 (3.6)	^1^*P* < 0.001

*Significantly different at *P* < 0.05; ^1^Mann-Whitney U test

### Logistic regression analyses of sexual assault

Bivariate analyses found that older age (Odds Ratio, OR = 1.04, CI = 1.00–1.07), being recruited in the general out-patient department (OR = 2.57, CI = 1.37–5.83), greater number of children (OR = 1.57, CI = 1.14–2.15), high psychological abuse (OR = 5.83, CI = 2.67–12.74), high physical violence (OR = 4.55, CI = 2.13–9.75), high danger (OR = 8.49, CI = 3.75–19.18) and suicidal behaviour in the past 12 months (OR = 4.58, CI = 2.16–9.74) were associated with sexual assault. In multivariable logistic regression high psychological violence (OR = 3.42 (1.07–11.01) was statistically significant and high physical violence (OR = 2.42, CI = 0.95–6.16) and suicidal behaviour (OR = 2.62, CI = 0.94–7.31) approached statistical significance with sexual assault (see Table 4).

**Table 4:** Predictors of sexual assault

Variable	UOR (95% CI)	P-value	AOR (95% CI)^a,b^	P-value
Demographics and partner characteristics				
Age in years	1.04 (1.00–1.07)	0.033	–	
Formal education				
Primary or less	1 (Reference)		–	
Secondary	0.55 (0.27–1.12)	0.099		
Post secondary	0.71 (0.30–1.70)	0.446		
Recruitment				
Antenatal care clinic	1 (Reference)		1 (Reference)	
General out-patient clinic	2.57 (1.37–5.83)	0.003	1.46 (0.57–3.75)	0.431
Number of children	1.57 (1.14–2.15)	0.006	1.31 (0.79–2.19)	0.294
Currently employed	1.27 (0.64–2.51)	0.493	–	
Economic household situation				
Low	1 (Reference)		–	
High	0.94 (0.50–1.78)	0.848		
Marital status				
Married/cohabitating	1 (Reference)		–	
Single/divorced/separated	1.60 (0.26–1.40)	0.239		
Currently living with the perpetrator	0.70 (0.31–1.61)	0.400	–	
Left partner in the past 12 months	1.77 (0.65–4.82)	0.264	–	
Other types of intimate partner violence				
Psychological abuse				
Low (0–2)	1 (Reference)		1 (Reference)	
Medium (3–6)	0.99 (0.40–2.46)	0.979	0.74 (0.27–2.07)	0.568
High (7 or more)	5.83 (2.67–12.74)	< 0.001	3.42 (1.07–11.01)	0.039
Physical violence				
Low (0–1)	1 (Reference)		1 (Reference)	
Medium (2–6)	1.83 (0.77–4.31)	0.170	1.80 (0.69–4.93)	0.233
High (7 or more)	4.55 (2.13–9.75)	< 0.001	2.42 (0.95–6.16)	0.064
Danger				
Low (0–1)	1 (Reference)		1 (Reference)	
Medium (2–3)	3.13 (1.33–7.38)	0.009	1.16 (0.38–3.55)	0.794
High (4 or more)	8.49 (3.75–19.18)	< 0.001	2.03 (0.65–6.36)	0.226
Suicidal behaviour in the past 12 months	4.58 (2.16–9.74)	< 0.001	2.62 (0.94–7.31)	0.065

*UOR* Unadjusted Odds Ratio, *AOR* Adjusted Odds Ratio, *CI* Confidence Interval

^a^Logistic regression, using forced entry; ^b^Hosmer & Lemeshow Chi-square = 9.96, *P* = 0.268; Nagelkerke R^2^: 0.30

## Discussion

This is the first study in Thailand screening a large sample of women for intimate partner violence and provides prevalence rates of different types of intimate partner violence including sexual assault in a sample of women attending a health care setting service. From 14,288 women screened in antenatal care and in general out-patient clinics in hospitals, 1.5% screened positive for the past 12 months intimate partner violence. This prevalence seems to be comparable with similar studies in similar settings (2.2% physical abuse in six European countries) [4] and Asian countries (ranging from 1.2% in Japan to 4.0% in Thailand) [5]. In some local surveys in Thailand the prevalence of past 12 month intimate partner violence ranged from 4.8 to 26.6% [6–8]. One possible explanation for the higher prevalence in the latter surveys is that detailed validated intimate partner violence scales were used, while our intimate partner violence prevalence was obtained on the basis of two screening questions that did not include the range of women’s experiences of intimate partner violence [16].

Further, this study reports on the co-occurrence of different types of intimate partner violence, and describes the association between past 12 months intimate sexual victimization experiences with demographic variables, partnership characteristics, suicidal behaviour and hazardous alcohol use. In this study of women with intimate partner violence attending antenatal care or general hospital out-patient services a moderate prevalence of sexual assault was found, which seem lower than in similar previous studies [2, 3, 17–19]. The joint occurrence of psychological abuse, physical and sexual violence found in this study seemed also lower than in some previous studies [3]. The overlap between different types of intimate partner violence that often occur simultaneously seem to “confirm that intimate partner violence are often part of a broader pattern of controlling behaviour” [20]. In agreement with previous studies [14, 21, 22], in this study sexually assaulted women were also more likely to report threatening or attempting suicide. This finding highlights the importance of mental health management, particularly regarding suicidal behaviour, and history of sexual assault [23].

The investigation found that in bivariate analysis that of the types of violence (psychological abuse, physical violence and danger) and in multivariable analysis that physical violence was associated with sexual assault. Similar results were found in previous studies [3, 12, 18, 24, 25]. McFarlane et al. [12] argue that higher scores of psychological abuse and physical violence for sexually assaulted women support a continuum of aggression theory. In particular, specific physical and psychological violent behaviour such as an increase in severity or frequency of physical violence, the use of a weapon, threat to kill, choking, illegal drug use, violence and constantly jealous, control of daily activities, spying, and having been beaten while pregnant were found in this study to be more frequent among sexually assaulted compared to non-sexually assaulted women. Information on acts of physical and psychological violence that are more likely to co-occur with sexual assault has important implications for legal investigations [24].

In bivariate analysis, the study found, in agreement with other studies [2, 21, 22] that sexually assaulted women reported statistically significant more risk factors for danger or femicide. Of concern was the finding that almost half of sexually assaulted women had received death threats by their intimate partners. The study found a high prevalence of hazardous drinking or having an alcohol use disorder, but this was not associated with sexual assault victimization, as found in some previous studies [2, 12]. A few of the women had left the partner in the past 12 months, which seemed not to have significantly reduced sexual intimate partner violence.

### Study limitations

The results of this study cannot be generalized to all female survivors of intimate partner violence in Thailand since the current study recruited antenatal care and general out-patients in one region. Further, the study relied on self-reports which may under- or over-report due to lack of adequate recall or lack of voluntary disclosure [2]. Sexual assault was assessed in face-to-face interviews, which may have led to more under-reporting than in self-report questionnaires [26]. Some other studies [8, 20, 26] have found that the assessment of injury resulting from intimate partner violence and the experience of early violence (such as childhood sexual abuse) added an important dimension of intimate partner violence, which should be added in future research.

## Conclusions

The study found a moderate rate of sexual assault in intimate violent partner relationships and those sexual assaults are more likely to co-occur with physical intimate partner violence and suicidal behaviour. This knowledge may be helpful in the detection and management of sexual assault in intimate violent partner relationships of women in health care settings in Thailand.

## Abbreviations

AUDIT-C: Alcohol Use Disorder Identification Test– Consumption
IPV: Intimate Partner Violence
OSCC: One-Stop Crisis Centre
SVAWS: Severity of Violence Against Women Scale
